# Parenchymal Cell Damage and Bile Duct Activity in the Precancerous Liver

**DOI:** 10.1038/bjc.1962.73

**Published:** 1962-12

**Authors:** P. M. Sutton

## Abstract

**Images:**


### PARENCHYMAL CELL DAMAGE AND BILE DUCT ACTIVITY IN THE PRECANCEROUS LIVER

P. M. SUTTON

*From the Department of Morbid Anatomy, University College Hospital Medical School, London, W.C.1.*

Received for publication August 16, 1962

STUDY of the effects of toxic agents on the liver has given us a considerable body of knowledge about the histological and biochemical abnormalities resulting from the action of specific drugs. Less is known about what happens if these are given concurrently nor do we know whether their individual effects on hepatic tissue are retained or merged with one another. Such studies might well supply new data concerning both their modes of action and also the reactions of liver cells to damage, in the same way as Rous and Larimore's (1920) experiments combining portal vein ligation with bile duct obstruction produced a result unpredictable from the known effects of ligation of each separately.

In a previous paper (Sutton, 1960) the combination of carbon tetrachloride ($CCl_4$) and $\alpha$-naphthyl-isothiocyanate (ANI) was used. This gave disappointing results for there was merely a summation of their individual effects. The present paper describes the action of repeated doses of $CCl_4$ on the rat liver already undergoing carcinogenesis by feeding with *p*-dimethylaminoazobenzene (butter yellow, DAB). In view of the known association between cirrhosis of the liver and hepatic cancer, it was suspected that $CCl_4$, which produces liver necrosis as an acute effect and cirrhosis as a chronic, might alter the pattern of carcinogenesis induced by DAB. Also, unlike ANI which acts almost entirely on the bile ducts, both $CCl_4$ and DAB affect the hepatic parenchymal cells, and it was wondered how they would respond to this double injury. In a paper dealing mainly with the effects of different diets on liver tumours induced by DAB, Miller *et al.* (1941) reported that subcutaneous injections of $CCl_4$ had no obvious effect on liver tumour production (though $CCl_4$ given prior to DAB may have slightly increased the rate of tumour formation).

### MATERIALS AND METHODS

One hundred and three adult Wistar albino rats of both sexes were used, the average weight at the start of the experiment was 150 g. The animals were split into three groups: 45 rats received both DAB and $CCl_4$, 38 DAB alone and 20 $CCl_4$ alone. DAB was mixed with MRC powder 41 in a concentration of 0·06 per cent. $CCl_4$ was given by Forbes' (1939) inhalation method once a week commencing two weeks after the rats had started DAB feeding, and continued for 24 weeks except for a gap of 3 weeks following the 14th week when no $CCl_4$ was given, though DAB feeding continued. All animals had access to unlimited water. Rats were weighed regularly and killed by excess ether at appropriate intervals throughout the experiment up to 36 weeks; if they were receiving both DAB

and $CCl_4$ they were killed 7 days after the last inhalation of $CCl_4$. Six rats died and were useless for histology because of autolysis. Careful post mortem examinations were made and material taken for histology from liver, spleen, lung and kidney as a routine, together with any other organs appearing abnormal. Sections were cut at 5 $\mu$, and stained with Ehrlich's acid haematoxylin and eosin (H. and E.), Van Gieson's method, silver impregnation for reticulin and periodic-acid-Schiff (PAS).

### RESULTS

Weekly inhalations of $CCl_4$ administered to rats already on a DAB-containing diet produced a marked contrast in the early histological changes in the liver by comparison with those induced by DAB alone. When DAB alone is fed, changes occur in both the biliary system and the hepatic parenchyma. The earliest changes are those of bile duct proliferation, which may be extreme giving a cirrhotic-like histological picture plus certain focal bile duct lesions which will be considered in more detail below. Meanwhile, the parenchymal cells undergo progressive alterations in their nuclei and cytoplasm, with the eventual appearance of nodules of hyperplastic liver cells. Finally, after about 6 months malignant tumours arise, the majority of parenchymal cell origin but with some cholangiocarcinomas. The essential differences between the two main groups of rats, those on DAB plus $CCl_4$ and those on DAB alone were of a quantitative rather than a qualitative nature. Since no special histological features were discovered which would warrant a detailed pathological description the results will be presented as a comparison between these two groups of animals, using the terminology employed by Opie (1944) and Firminger (1955) in their accounts of DAB carcinogenesis. Even inside each of the two main groups, considerable variation existed from animal to animal in the degree of hepatic change found in rats killed at the same time. Accordingly the findings will be given at three monthly intervals so that the groups are sufficiently big to make the results significant. The third group of rats receiving only weekly inhalations of $CCl_4$ showed the typical zonal areas of centrilobular liver necrosis 24 hours after administration of the toxic agent, with complete regeneration over the next 7 days before the next dose. When these rats were killed 10 days after the last of 24 weekly attacks of $CCl_4$ induced necrosis, their livers showed no abnormality and in particular no cirrhosis (Fig. 1). In the results described below animals on $CCl_4$ alone will not be considered further.

*Months* 1–3.—The most marked difference was the far greater degree of bile duct activity found in animals on DAB plus $CCl_4$ compared with those on DAB alone. About one half of the rats receiving the former treatment developed the striking lesion called by Opie (1944) cholangiofibrosis (Table I). Here foci of hyperplastic bile ducts with frequent mitoses lay in a cellular stroma of young

TABLE I.—*Incidence of Cholangiofibrosis (C.F.) in Rats*

| | DAB + $CCl_4$ | | DAB only | |
|---|---|---|---|---|
| Months | Total | With C.F. | Total | With C.F. |
| 1–3 | 15 | 7 | 14 | 0 |
| 4–6 | 12 | 10 | 12 | 1 |
| 7–9 | 14 | 12 | 10 | 7 |

fibroblasts and capillaries (Fig. 2). Often a column of bile duct cells enclosed collections of PAS-positive mucus, containing small groups of polymorphs whilst the whole lesion was infiltrated with plasma cells. On purely histological grounds the early nodules of cholangiofibrosis do resemble malignant tumours of bile ducts but this question will be taken up later in this paper. No cholangiofibrosis was found at this stage in rats on DAB alone and because these cholangiofibrotic areas are easily seen macroscopically as depressed white scars often near the liver surface, as Gupta (1956) has emphasized, it is unlikely that any were overlooked at autopsy. Together with this increase in cholangiofibrosis, animals receiving both drugs showed a greater degree of bile duct proliferation sometimes in adenomatous groups such as Orr (1940) described, and even in those rats without such extreme bile duct activity there was a greater degree of proliferation of small, dark-staining spindle-shaped cells called by Farber (1956*b*) " oval cells " and probably of biliary origin, extending deep into the lobules from the portal tracts. By contrast, rats on DAB only showed slighter bile duct activity and fewer oval cells extending from portal areas (Fig. 3). However, no significant difference was detected in the parenchymal cells between the two groups. Both showed small islands of enlarged liver cells with loss of cytoplasmic basophilia and nuclei up to double their normal size with prominent nucleoli and occasional cell divisions, though no abnormal mitotic figures were found. Almost no necrosis was detected in rats on DAB alone ; an occasional cell with brightly eosinophilic cytoplasm and a pyknotic nucleus was seen (individual cell necrosis) but nothing more. No animals in either group developed malignant tumours during this period.

*Months* 4–6.—By now the majority of rats receiving DAB with $CCl_4$ had developed cholangiofibrosis, whereas only one of the animals receiving DAB alone showed this lesion (Table I). At this time these areas of cholangiofibrosis were larger and the more central bile ducts were less hyperplastic with fewer mitoses and less hyperchromatic than the foci of cholangiofibrosis found during the first three months (Fig. 4). In places this trend had continued to a dense fibrosis in the middle of the lesion (Fig. 5), with acellular collections of mucus and small islands of hepatic cells trapped in the fibrous tissue. Once again the animals receiving both toxic agents showed the greater degree of bile duct activity and again there was no difference in the appearance of the parenchymal cells between the two groups. By this time both sets of animals had nodules of actively growing liver cells, with great nuclear variation and quite frequent cell divisions in which occasional abnormal mitoses, in the form of chromosomal bridges, were found. In no case were these nodules sufficiently large or atypical enough to be classified as malignant tumours. A few rats on DAB plus $CCl_4$, but none on DAB alone, had enough fibrous tissue formation accompanying the bile duct proliferation, with sufficient distortion of the hepatic architecture on Van Gieson and reticulin staining to warrant the description of portal cirrhosis.

*Months* 7–9.—From the 24th week until the 36th week when the experiment was concluded the animals in both groups developed malignant tumours of the liver. No metastases were detected, but this was probably because the rats were killed once an abdominal mass was palpated. No significant difference was detected in either the induction time or in the final incidence of liver cancer between the two groups, 11 out of 14 rats on DAB plus $CCl_4$ compared with 9 out of 10 rats on DAB alone developing malignant tumours. At this stage, as Table I

shows, there was now little difference in the incidence of cholangiofibrosis though these areas were usually larger in animals exposed to both drugs.

Regarding the type of liver tumour induced the histological picture showed so much variability in different areas from the same liver that classification was not easy. In general terms, the following conclusions were drawn :

(a) In both groups the commonest tumour was one showing a pleomorphic pattern ranging from hepatocarcinoma to the adenohepatoma of other authors.

In a minority there was a definitely cholangiocarcinomatous component. All the tumours showed much necrosis, frequent mitoses with many abnormal forms and tumour giant cells.

(b) Some tumours were of a purely hepatocellular type with a pattern similar to that of embryonic liver and including small islands of haemopoiesis. No bile secretion was detected. These tumours sometimes developed in livers containing nodules of cholangiofibrosis and were so morphologically different from the latter that it was difficult to conceive that they had arisen from them. On the other hand, in rats on DAB alone frank hepatocarcinomas were found in 2 animals in which no cholangiofibrosis at all was present (Fig. 6).

(c) A minority of the tumours were of a purely cholangiocarcinomatous pattern (Fig. 7). In at least two rats the histology strongly suggested that a cholangiocarcinoma had arisen from an area of cholangiofibrosis.

*Other findings.*—In both groups the spleen was enlarged to several times the normal size ; microscopy showed extreme congestion of the red pulp with large islands of haemopoietic cells. A few rats in both groups developed a chronic inflammatory lesion in the caecum similar to that described by Orr (1940). Rats on DAB alone gained more weight (mean +110 g.) than those on DAB plus $CCl_4$ (mean +60 g.).

### DISCUSSION

In experimental work on liver tumours the nature of the lesion termed cholangiofibrosis has been disputed. The word was coined by Opie (1944), who implied a precancerous condition in his account of the pathogenesis of the tumours produced by DAB ; a similar view was taken by Gupta (1956) in studies of the effect of thioacetamide on rat liver. Price *et al.* (1952), describing the progressive histological alterations occurring after azo-dye feeding found that regardless of type, liver tumours always arose in an area of cholangiofibrosis. However, Fir-

---

### EXPLANATION OF PLATES

FIG. 1.—Normal liver from $CCl_4$ control, 10 days after 24 weekly inhalations of $CCl_4$. H. and E. ×184.

FIG. 2.—Area of cholangiofibrosis showing hyperplastic biliary epithelium around a central plug of mucus and polymorphs. Nine weeks DAB plus $CCl_4$. H. and E. ×184.

FIG. 3.—Early bile duct and " oval " cell proliferation but no cholangiofibrosis. Nine weeks DAB alone. H. and E. ×184.

FIG. 4.—Larger area of cholangiofibrosis. Fourteen weeks DAB plus $CCl_4$. H. and E. ×184.

FIG. 5.—Central portion of a cholangiofibrotic nodule showing small clumps of hepatic parenchymal cells in relatively acellular fibrous tissue. H. and E. ×175.

FIG. 6.—Hepatocarcinoma, showing a sheet of uniform cells of parenchymal cell origin with mitotic figures. Thirty-five weeks DAB only. H. and E. ×385.

FIG. 7.—Cholangiocarcinoma with an alveolar pattern and tumour giant cells. Thirty-two weeks DAB plus $CCl_4$. H. and E. ×385.

1 2 3 4

Sutton.

5

6

7

Sutton.

minger's (1955) experience of both DAB and 2-acetylaminofluorene (AAF) was that although cholangiocarcinomas did arise in areas of cholangiofibrosis, yet they comprised only a small minority of the induced liver tumours. Farber (1956*a*), using ethionine, noted the histological simulation of cancer by areas of cholangiofibrosis but believed that these were not true tumours. On the other hand, both Orr (1940) and Grant and Rees (1958) considered this lesion a frank cholangiocarcinoma ; whilst White and Edwards (1942) argued that it was not a tumour at all, because it was not transplantable, it neither invaded blood vessels nor metastasised and progressed through atrophic changes to eventual fibrosis. Popper and Schaffner (1957), in their review agree that cholangiofibrosis should not be regarded as neoplastic.

It appears from the experiments reported here on combined DAB and $CCl_4$ intoxication that cholangiofibrosis is not a necessary precursor of cancer of the liver because (a) some animals developed malignant tumours in the absence of this lesion, and (b) the increased induction of cholangiofibrosis by DAB plus $CCl_4$ was not associated with an increased production of liver cancer. However, in a minority of the animals frank cholangiocarcinomas did arise in areas of cholangiofibrosis.

Grant and Rees (1958) described an increased incidence of cholangiocarcinoma (this is their terminology for what is here called cholangiofibrosis ; in what follows the term cholangiofibrosis will be used for the sake of clarity) in rats fed DAB and a normal diet compared with another group given DAB plus supplemented B vitamins. In their description of the early changes in the liver much more parenchymal cell necrosis was observed in those animals without added vitamins which developed cholangiofibrosis. This is interesting since in the present experiment the rats given DAB plus $CCl_4$ which showed the earliest and greatest incidence of cholangiofibrosis also had more parenchymal cell necrosis due to the acute effects of $CCl_4$. In the present investigation it is not yet known whether the necrosis induced by $CCl_4$ or the amounts of B vitamins ingested were of greater importance. Hypovitaminosis cannot be excluded, especially as the animals receiving both toxic compounds gained less weight than those fed DAB alone.

It is not immediately obvious why the addition of repeated doses of $CCl_4$ to animals already receiving DAB should affect the bile ducts and connective tissue of the portal tracts rather than the parenchymal cells. The problem is similar to the classical one of chronic $CCl_4$ poisoning and subsequent portal cirrhosis where repeated centrilobular necrosis gives rise to cirrhosis (Cameron and Karunaratne, 1936). As Cameron and Karunaratne observed, if sufficient time elapses between the $CCl_4$ injections for the liver to recover (i.e. to regenerate and fill up the necrosed zones with new cells) then no cirrhosis results however long the experiment is continued. In this present work control rats given weekly $CCl_4$ for 24 weeks did not develop cirrhosis, whereas previously (Sutton, 1960) rats given approximately the same amount of $CCl_4$ twice weekly developed the histological picture of portal cirrhosis by 12 weeks. Cameron and Karunaratne suggested that the cirrhosis was due to stimulation of the periportal fibrous tissue by the passage there of the products of autolysis from the necrotic centrilobular zones.

When liver tissue is lost, e.g. by partial hepatectomy, there is evidence that the residue is stimulated into active regeneration either by the drop in concentration of a circulating tissue-specific growth inhibitor (Weiss and Kavanau, 1957 ;

Glinos, 1958) or by the rise in blood levels of growth stimulating factors (Cameron, 1955; Weinbren, 1959). Abercrombie and Harkness (1951) have shown that following partial hepatectomy parenchymal cells undergo a burst of mitotic activity, maximum at 24 hours, followed at 48 hours by a second smaller peak in bile duct epithelium, sinusoidal cells and probably fibroblasts around portal tracts. This suggests strongly that these non-parenchymal liver cells are also regulated by factors similar to those that control the growth of hepatic parenchymal cells, but that their response is neither so great nor so rapid as the latter. Abercrombie (1957) thinks that this involves the existence of definite organ races of connective tissue cells and quotes the work of Grobstein (1953) on tissue culture studies on embryonic induction in support of his hypothesis.

This proliferation of portal tract tissue after hepatectomy suggests an alternative explanation to that of a stimulation by autolytic tissue for the production of cirrhosis by $CCl_4$: that the sustained lack of liver tissue will result in prolonged specific activation of the non-parenchymal hepatic cells by the circulating factors, thus giving the mixture of fibroblastic, vascular and biliary proliferation found in cirrhosis. Such a situation arises with twice weekly $CCl_4$ intoxication whereas with less frequent doses the liver is able to regenerate completely and the circulating factors return to normal and the less responsive non-parenchymal cells are not stimulated. This model explains why the earliest proliferative changes in portal cirrhosis are periportal since the circulating factors reach this zone of the liver lobule first. It can possibly account for the portal cirrhosis found after prolonged fatty infiltration of the liver, in which necrosis is absent, by assuming that the degenerate fatty cells fail either to produce or to respond to the circulating factors, so that a slow proliferative response is provoked in the non-parenchymal elements. Perhaps, too, when liver carcinogens act on parenchymal cells with the eventual production of carcinomas a similar interference with the growth controlling mechanisms results so that the initial bile duct proliferation is an indicator of this primary action. Farber (1956*b*) has shown that the earliest changes found in the livers of rats fed either DAB, AAF or ethionine are bile duct and "oval" cell proliferation (the latter of probable biliary origin) without parenchymal cell necrosis. Many workers (e.g. Miller and Miller, 1953) have suggested that carcinogens act through damage to the specific cellular mechanisms controlling growth. The idea here is that the initial excessive bile duct activity found by giving $CCl_4$ together with DAB is accounted for by both substances affecting these growth controlling mechanisms in different ways, $CCl_4$ by producing necrosis and DAB by specific damage to these mechanisms short of actual cell death.

### SUMMARY

1. Rats were exposed to weekly inhalations of $CCl_4$ whilst receiving DAB in their diets over a period of months.

2. This resulted in much greater bile duct proliferation and the earlier appearance of cholangiofibrosis than found in rats on DAB alone; but no difference in either the parenchymal cells or in the final production of tumours. Some hepatocarcinomas arose in livers in which no cholangiofibrosis was present. Therefore, in these experiments cholangiofibrosis did not appear to be an essential precursor of liver cancer, although a few instances of cholangiocarcinomas arising in areas of cholangiofibrosis were found.

3. The excessive bile duct activity in rats fed DAB, following weekly $CCl_4$-induced parenchymal cell necrosis, is compared with the portal cirrhosis found with chronic $CCl_4$ intoxication and the underlying mechanisms discussed.

I wish to thank Sir Roy Cameron for constant help and advice during this work, Dr. J. F. Smith and Dr. W. G. Spector for helpful criticism of the manuscript and Miss Angela Cluff for skilled technical assistance throughout. Grateful acknowledgement is made to the British Empire Cancer Campaign for a grant covering the expenses of this investigation. This work was carried out during the tenure of the Charles Bolton Fellowship.

### REFERENCES

Abercrombie, M.—(1957) *Symp. Soc. exp. Biol.*, **11**, 235.
*Idem* and Harkness, R. D.—(1951) *Proc. Roy. Soc. B.*, **138**, 544.
Cameron, G. R.—(1955) in 'Lectures on the Scientific Basis of Medicine'. London (Athlone Press), vol. 3, p. 52.
*Idem* and Karunaratne, W. A. E.—(1936) *J. Path. Bact.*, **42**, 1.
Farber, E.—(1956*a*) *Arch. Path.*, **62**, 445.—(1956*b*) *Cancer Res.*, **16**, 142.
Firminger, H. I.—(1955) *J. nat. Cancer Inst.*, **15**, 1427.
Forbes, J. C.—(1939) *J. Pharmacol.*, **65**, 287.
Glinos, A. D.—(1958) in ' Liver Function '. Edited by R. W. Brauer. Washington (American Institute of Biological Sciences), p. 425.
Grant, H. C. and Rees, K. R.—(1958) *Proc. Roy. Soc.* B., **148**, 117.
Grobstein, C.—(1953) *Science*, **118**, 52.
Gupta, D. N.—(1956) *J. Path. Bact.*, **72**, 415.
Miller, J. A. and Miller, E. C.—(1953) in ' Advances in Cancer Research '. Edited by J. P. Greenstein and A. Haddow. New York (Academic Press), vol. 1, p. 340.
*Idem*, Miner, D. L., Rusch, H. P. and Baumann, C. A.—(1941) *Cancer Res.*, **1**, 699.
Opie, E. L.—(1944) *J. exp. Med.*, **80**, 231.
Orr, J. W.—(1940) *J. Path. Bact.*, **50**, 393.
Popper, H. and Schaffner, F.—(1957) in ' Liver Structure and Function '. New York (McGraw-Hill), p. 597.
Price, J. M., Harman, J. W., Miller, E. C. and Miller, J. A.—(1952) *Cancer Res.*, **12**, 192.
Rous, P. and Larimore, L. D.—(1920) *J. exp. Med.*, **31**, 609.
Sutton, P. M.—(1960) *J. Path. Bact.*, **79**, 157.
Weinbren, K.—(1959) *Gastroenterology*, **37**, 657.
Weiss, P. and Kavanau, J. L.—(1957) *J. gen. Physiol.*, **41**, 1.
White, J. and Edwards, J. E.—(1942) *J. nat. Cancer Inst.*, **3**, 43.


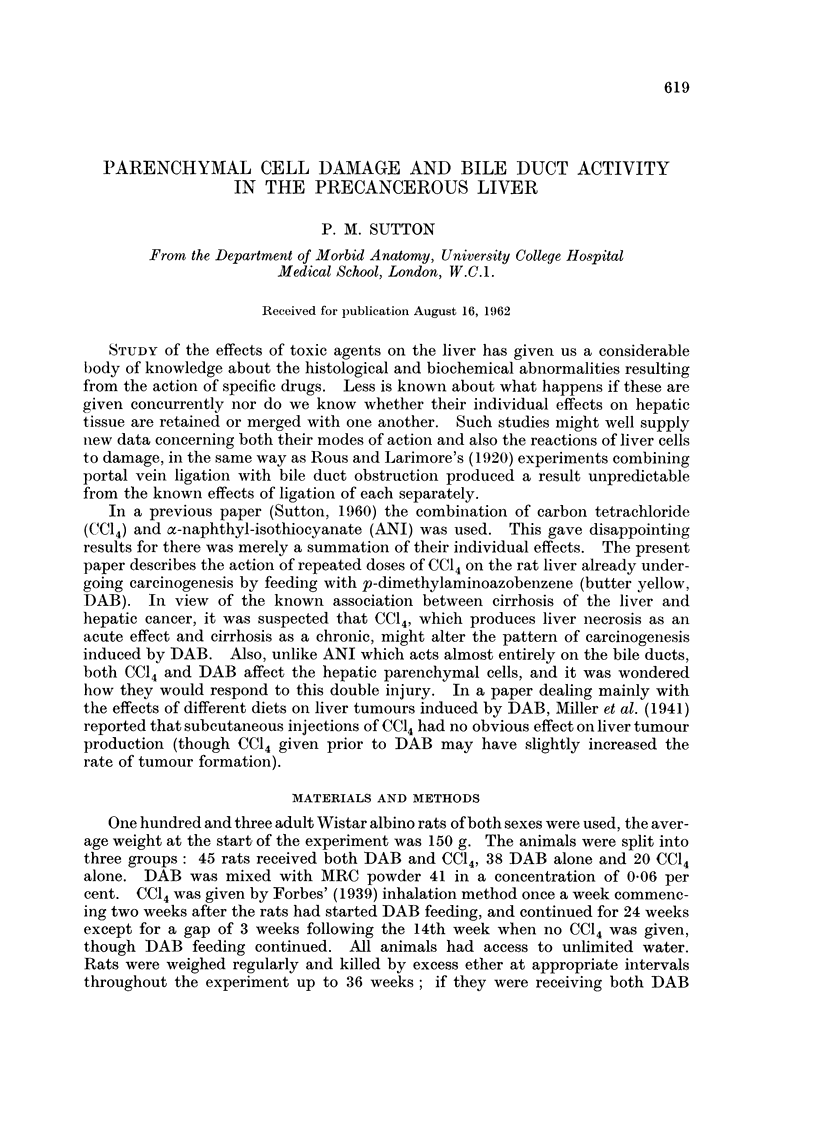

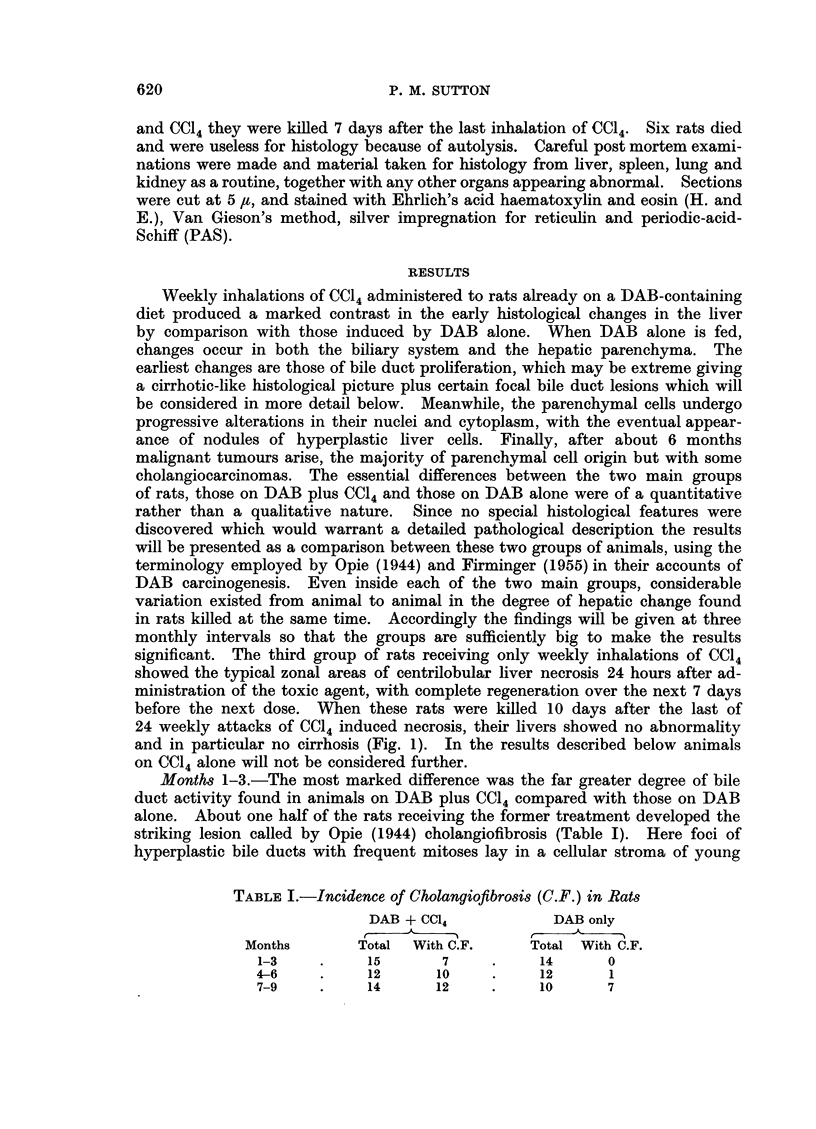

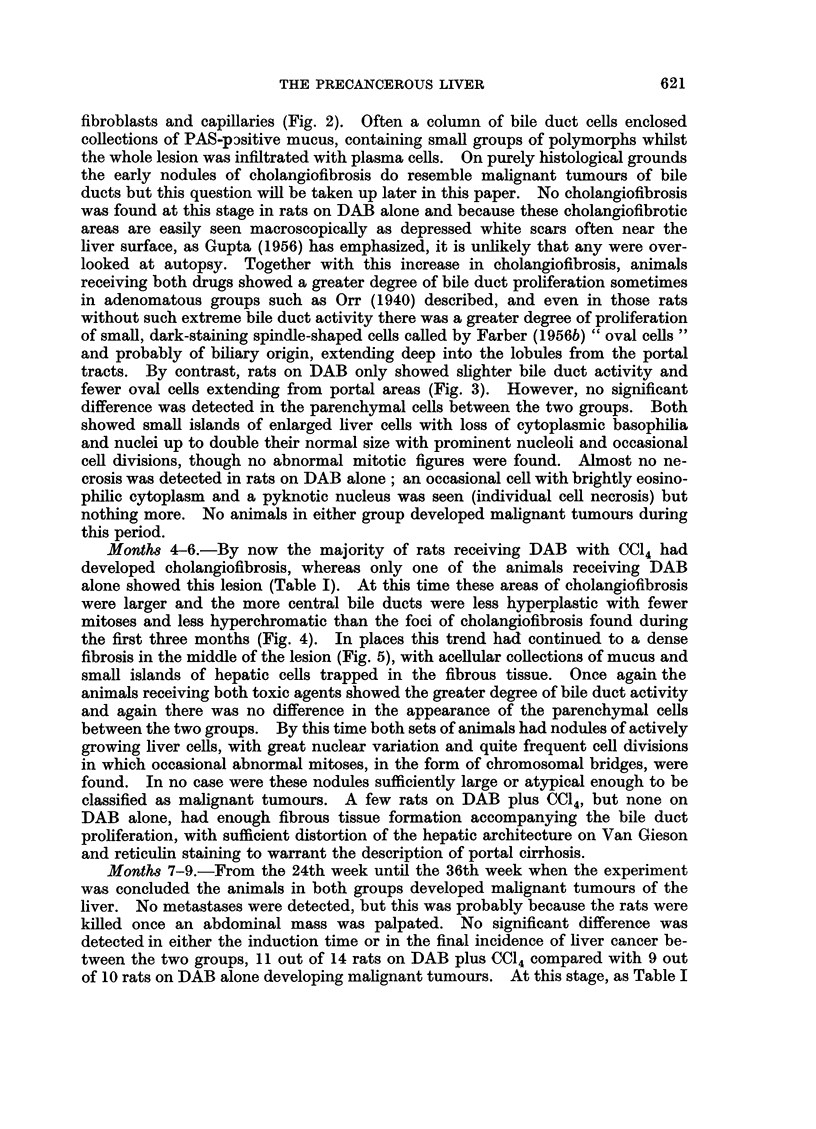

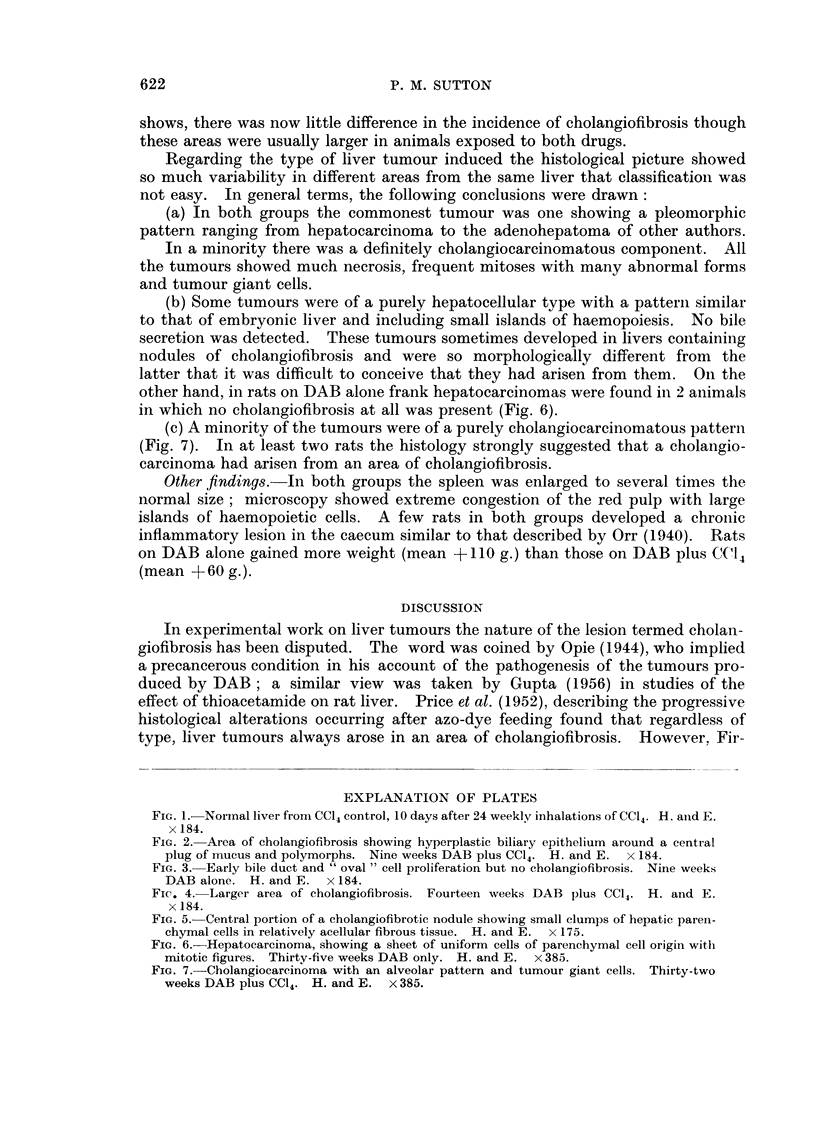

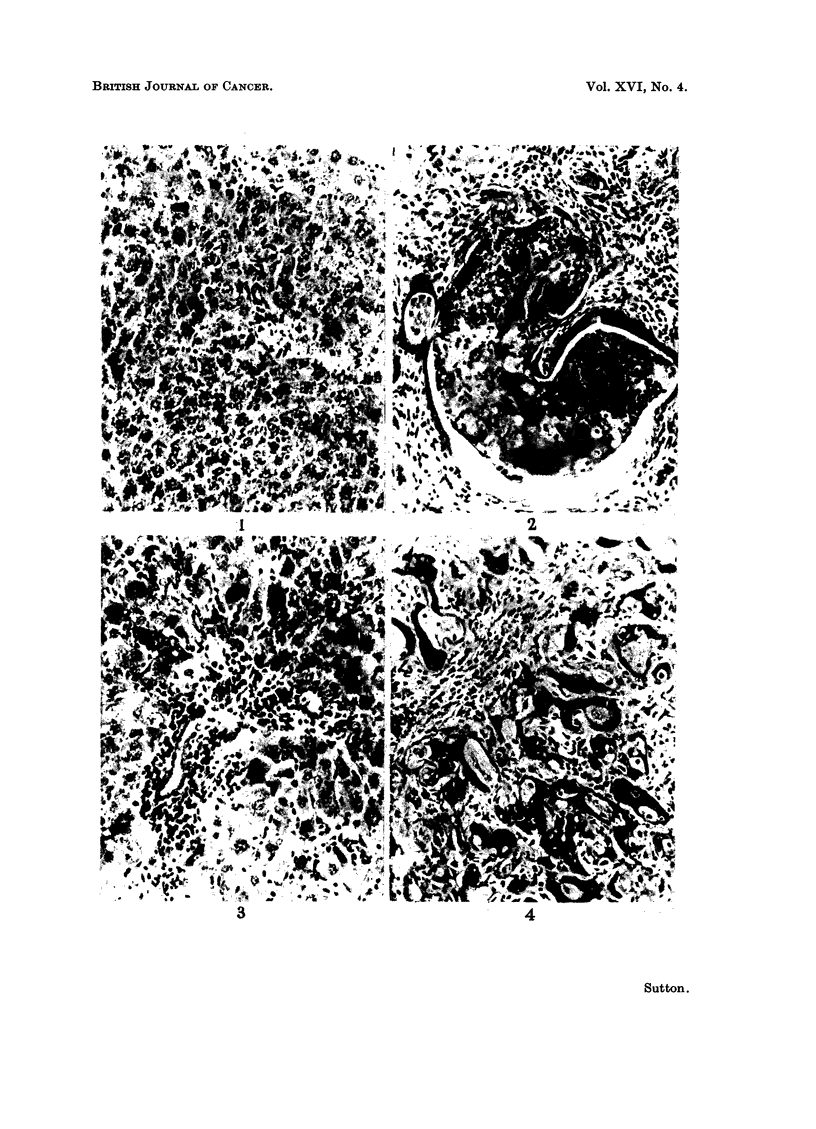

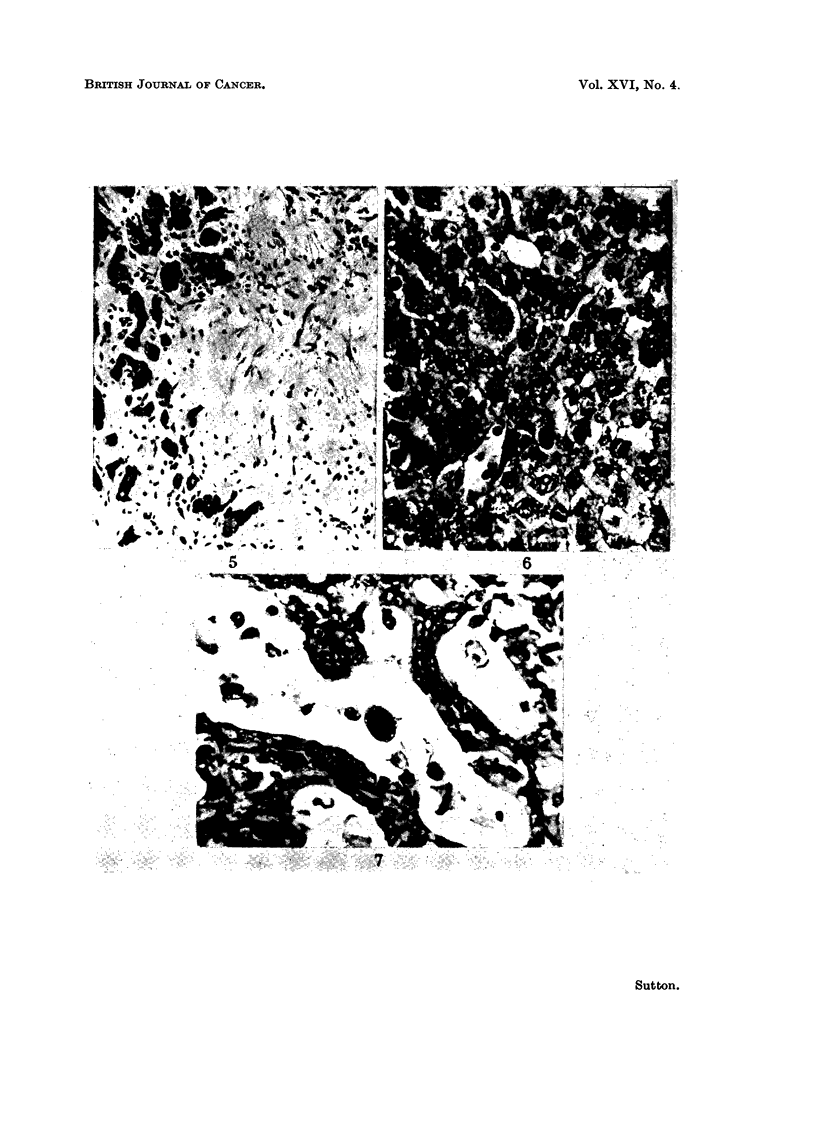

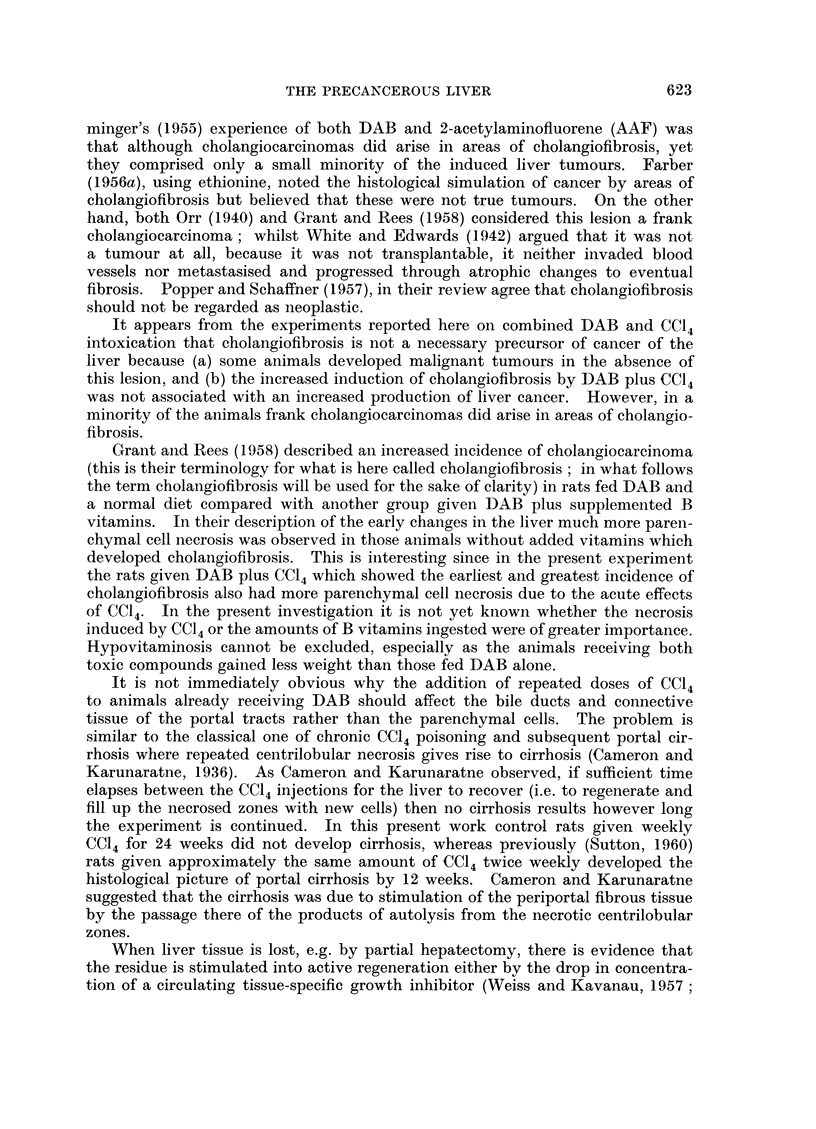

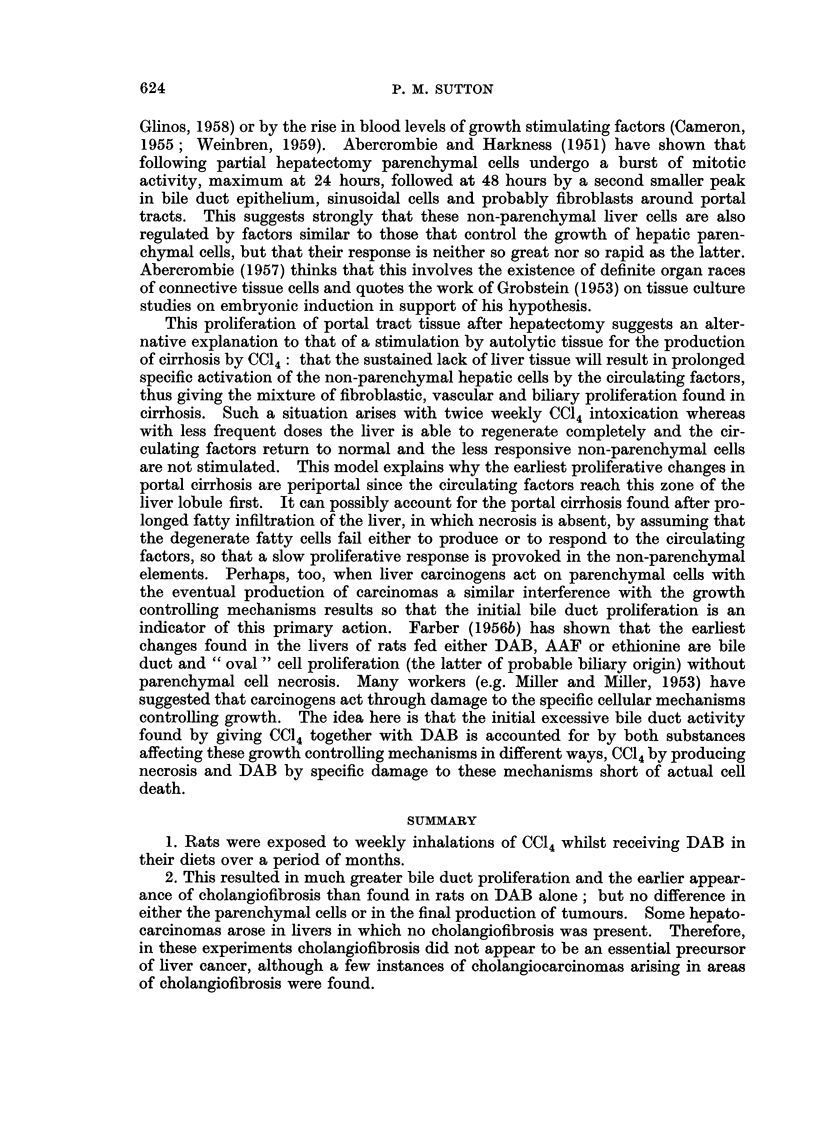

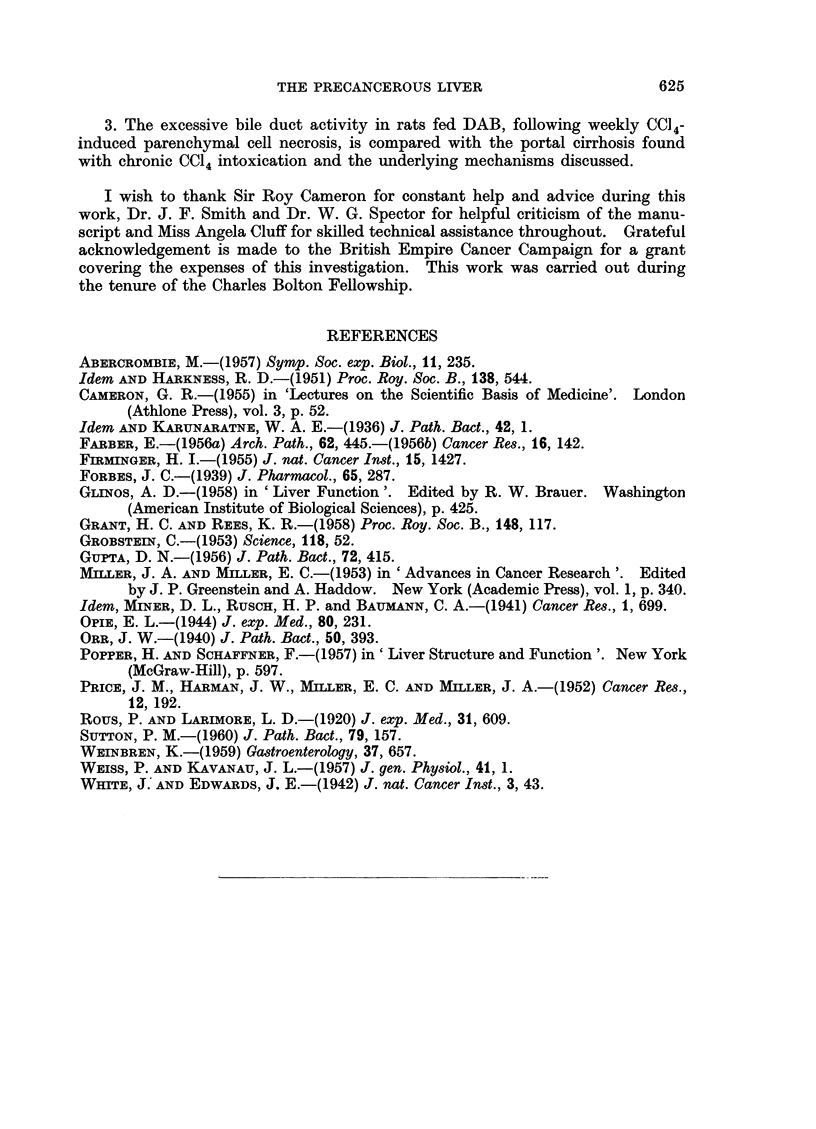


## References

[OCR_00392] ABERCROMBIE M., HARKNESS R. D. (1951). The growth of cell populations and the properties in tissue culture of regenerating liver of the rat.. Proc R Soc Lond B Biol Sci.

[OCR_00404] FIRMINGER H. I. (1955). Histopathology of carcinogenesis and tumors of the liver in rats.. J Natl Cancer Inst.

[OCR_00411] GRANT H. C., REES K. R. (1958). The precancerous liver; correlations of histological and biochemical changes in rats during prolonged administration of thioacetamide and butter yellow.. Proc R Soc Lond B Biol Sci.

[OCR_00427] PRICE J. M., HARMAN J. W., MILLER E. C., MILLER J. A. (1952). Progressive microscopic alterations in the livers of rats fed the hepatic carcinogens 3'-methyl-4-dimethylaminoazobenzene and 4'-fluoro-4-dimethylaminoazobenzene.. Cancer Res.

[OCR_00434] WEINBREN K. (1959). Regeneration of the liver.. Gastroenterology.

